# Virulence and Metabolism Crosstalk: Impaired Activity of the Type Three Secretion System (T3SS) in a *Pseudomonas aeruginosa* Crc-Defective Mutant

**DOI:** 10.3390/ijms241512304

**Published:** 2023-08-01

**Authors:** Teresa Gil-Gil, Trinidad Cuesta, Sara Hernando-Amado, Jose Antonio Reales-Calderón, Fernando Corona, Juan F. Linares, José L. Martínez

**Affiliations:** 1 Departamento de Biotecnología Microbiana, Centro Nacional de Biotecnología, CSIC, Darwin 3, Cantoblanco, 28049 Madrid, Spain; 2 Departamento de Microbiología II, Facultad de Farmacia, Universidad Complutense de Madrid, 28040 Madrid, Spain

**Keywords:** type III secretion, *Pseudomonas aeruginosa*, Crc, catabolic repression, metabolism-virulence crosstalk, proton motive force

## Abstract

*Pseudomonas aeruginosa* is a ubiquitous nosocomial opportunistic pathogen that harbors many virulence determinants. Part of *P. aeruginosa* success colonizing a variety of habitats resides in its metabolic robustness and plasticity, which are the basis of its capability of adaptation to different nutrient sources and ecological conditions, including the infected host. Given this situation, it is conceivable that *P. aeruginosa* virulence might be, at least in part, under metabolic control, in such a way that virulence determinants are produced just when needed. Indeed, it has been shown that the catabolite repression control protein Crc, which together with the RNA chaperon Hfq regulates the *P. aeruginosa* utilization of carbon sources at the post-transcriptional level, also regulates, directly or indirectly, virulence-related processes in *P. aeruginosa*. Among them, Crc regulates *P. aeruginosa* cytotoxicity, likely by modulating the activity of the Type III Secretion System (T3SS), which directly injects toxins into eukaryotic host cells. The present work shows that the lack of Crc produces a Type III Secretion-defective phenotype in *P. aeruginosa*. The observed impairment is a consequence of a reduced expression of the genes encoding the T3SS, together with an impaired secretion of the proteins involved. Our results support that the impaired T3SS activity of the *crc* defective mutant is, at least partly, a consequence of a defective protein export, probably due to a reduced proton motive force. This work provides new information about the complex regulation of the expression and the activity of the T3SS in *P. aeruginosa*. Our results highlight the need of a robust bacterial metabolism, which is defective in the *∆crc* mutant, to elicit complex and energetically costly virulence strategies, as that provided by the T3SS.

## 1. Introduction

*Pseudomonas aeruginosa* is a Gram-negative opportunistic pathogen that produces a wide range of nosocomial infections [1,2]. In addition, it is a major cause of the chronic infections suffered by cystic fibrosis and chronic obstructive pulmonary disease patients [3,4]. The capacity of *P. aeruginosa* to produce an infection involves the production of virulence factors, as well as a metabolic reorganization [1,5]. One of the global regulators of *P. aeruginosa* metabolism is the post-transcriptional catabolite repressor Crc. Together with the RNA chaperone Hfq, Crc forms a complex that bind its target mRNAs impeding their translation. Repression is retrieved by the small RNA *crcZ*, which competes with Crc/Hfq targets for their binding with the complex. This regulatory pathway allows the preferential use of carbon sources when *P. aeruginosa* grows in complex media [6,7], hence allowing the optimization of the metabolism and growth of the bacterial population [8,9].

In addition to its role in *Pseudomonas* catabolite repression, Crc is also responsible (either directly or indirectly) for the regulation of a large number of virulence-related processes, such as cell motility, biofilm formation, expression of quorum sensing-regulated virulence determinants and Type 3 Secretion (T3S) [10,11,12]. Moreover, Crc loss-of-function impairs *P. aeruginosa* virulence in *Dictytostelium discoideum* and modulates the susceptibility to antibiotics of this bacterial pathogen [10,13]. Therefore, Crc is a key element modulating the crosstalk among *P. aeruginosa* virulence, antibiotic resistance, and metabolism.

Among the different classes of secretion systems described in *P. aeruginosa* [14], the Type 3 Secretion System (T3SS) allows the injection of bacterial toxic effectors (ExoS, ExoT, ExoU, and ExoY) into the cytosol of eukaryotic cells [15]. It has been described that *P. aeruginosa* populations expressing functional T3SS during acute respiratory infections produce higher relapse rates and bacterial burden, leading to a six-fold increase in mortality of infected patients [16]. Conversely, it has been described that *P. aeruginosa* clones causing chronic infections in cystic fibrosis patients develop impaired T3S and quorum sensing phenotypes, during their inside-host evolution [17,18]. Therefore, *P. aeruginosa* presents a diversity of mechanisms, including T3S, whose activity may vary depending on the different stages of the infection and the different types of infections, allowing survival and persistence in varied habitats [19]. In fact, it has been described that T3S is beneficial or detrimental in different stages and types of infections [20].

The T3SS injection machinery consists of more than 25 proteins, organized in a complex syringe structure composed of three components: the basal body, the needle, and the translocon [15,21]. The basal portion of the injectisome consists of a needle-like structure composed of two groups of protein rings which may protrude from the basal surface up to 50–80 nm long [22,23,24]. The needle is the channel trough which effectors (exoproteins) travel in a polarized manner to reach the target cell and it also functions as a checkpoint, allowing only fully unfolded proteins to be secreted [24]. Upon host-cell contact, a connection between the needle tip complex and a pore formed in the host cell membrane is established, leading to the translocation of effectors [25]. Purified *P. aeruginosa* T3SS-needles are mostly composed of a helical PscF multimer, which brings effectors from the basal body to the translocator apparatus, so that mutants lacking this gene lose the ability to secrete the effector proteins [26]. Evidence obtained by studying T3SS in several bacteria suggests that translocators are exported before than the effector proteins. This translocator apparatus is made of two hydrophobic proteins, PopB and PopD, together with the hydrophilic protein PcvR, which is needed for the functional assembly of the translocon. PopB and PopD are needed to trigger effectors’ secretion upon contact with the host, suggesting that there is a secretion hierarchy [14,27,28]. It has been described that the association and insertion of the translocators into the membrane occurs as heterocomplexes, containing 8 molecules of each protein, which is a critical process during translocon assembly [29]. *exsA* encodes the master transcriptional activator of the expression of the genes encoding T3SS proteins, which is expressed under specific in vitro inducing conditions or upon contact with the host cells [30,31,32]. The expression of *P. aeruginosa* T3SS is regulated by a complex cascade of activators and anti-activators. ExsA is the master transcriptional activator of the system. Under non-induced conditions, ExsA is sequestered by ExsD, and hence the expression of T3SS genes is repressed. The next protein in the cascade is the anti-anti-activator ExsC. When this protein is present at high levels inside the cell, it binds ExsD, ExsA is present in its free, active form, and T3SS system expression is triggered. A main player in the system in ExsE. This protein binds ExsC, hence impeding its anti-anti-activator activity. However, when T3S is triggered, ExsE is exported, which is the first step in triggering the regulatory cascade [33]. In addition to this, the system presents other regulatory elements that allow, among other issues, a hierarchical regulation of the expression and the activity of the T3SS [34]. The regulatory network that controls T3S is also dependent on metabolism. Accordingly, the presence of tryptophan catabolites (IAA, NAA and 3-hydroxykynurenine), modulate the expression of T3SS-related encoding genes [35]. In addition, mutants in the *aceAB* operon, which encodes the pyruvate dehydrogenase [36], as well as strains overexpressing *hutT*, encoding an histidine transporter [37], are defective in T3S, hence supporting a linkage between this complex virulence determinant and the bacterial metabolism.

It is already known that a *crc* defective mutant modulates the generation of vesicles and secreted virulence determinants of *P. aeruginosa* and is less virulent and cytotoxic than its parental strain [10,11]. Moreover, it has been shown that a *crc*-deficient mutant presents impaired T3S [10] and it has been suggested that Crc positively controls, at the transcriptional level, the expression of the genes encoding the proteins that constitute the T3SS [38,39]. Finally, it has been previously shown that a proficient Proton Motive Force (PMF) is needed for the correct functioning of the T3SS [40,41]. In this work, we show that the lack of Crc compromises *P. aeruginosa* PMF and that this metabolic defect might be in the basis of the reduced T3S of the *crc* mutant.

## 2. Results

### 2.1. Lack of Crc Precludes the Injection of the T3SS Effector ExoS by P. aeruginosa in HeLa Cells

**Figure 1:**
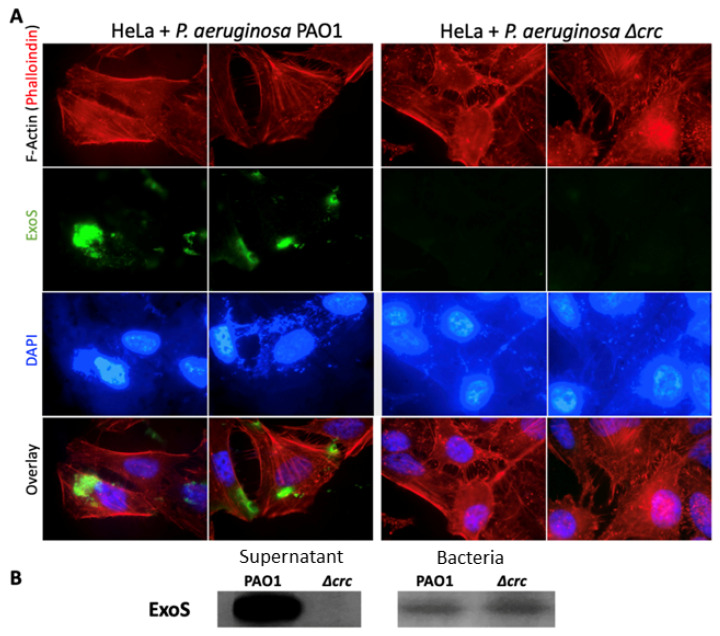
The amount of the T3SS effector ExoS injected into HeLa cells and its extracellular accumulation is defective in the *P. aeruginosa Δcrc* mutant (**A**) HeLa cells were infected with *P. aeruginosa* PAO1 and *Δcrc* at MOI of 50 and incubated during at 37 °C 3 h. Cells were fixed, and ExoS was visualized at 63x magnification by immunofluorescence (green), F-Actin with Phalloidin (red) and the DNA with DAPI (blue). (**B**) Bacterial extracts and supernatants from PAO1 and *Δcrc* strains obtained in T3SS-inducing conditions were analyzed by Western blot with an anti-ExoS antibody.

Previous work has shown that a *P. aeruginosa crc* deficient mutant is less virulent and cytotoxic, and presents impaired T3S than its parental counterpart [10,11]. However, the effect of Crc on the in vivo and production and secretion of the T3SS effectors has not been explored. To address this issue, the wild-type *P. aeruginosa* strain PAO1 (PAO001, Holloway strain) and its isogenic deletion mutants *Δhfq* (see below) and a previously constructed *Δcrc* mutant [11] were used. To note that the whole genome sequencing of the *Δcrc* mutant has been previously performed and it does not contain any other secondary mutation besides the deletion of *crc* [11]. HeLa cells were co-incubated with *P. aeruginosa* and the injection of the exotoxin S (ExoS) was visualized by confocal microscopy using a specific anti-ExoS antibody (Figure 1A). After 3 hours of infection, both strains were capable of adhering to the HeLa cells. The number of bacteria attached was slightly lower in the case of the *crc* mutant (69 ± 18%), this difference not being statistically significant. As shown in the Figure 1A, only the wild-type strain was able to inject the toxin inside the cell host, suggesting a defect on the secretion of ExoS in the *crc* mutant. To further confirm this possibility, ExoS secretion was also analyzed by Western blot, using an anti-ExoS antibody, in bacterial cultures grown under T3SS-inducing conditions. Consistent with the results from HeLa cells, ExoS was abundant in the culture supernatants of the wild-type strain, and it was not detected extracellularly in the *crc* defective mutant (Figure 1B). Overall, these results indicate that the *crc* defective mutant presents an impaired amount of extracellular ExoS, being this a possible cause for the impaired cytotoxicity of this mutant.

### 2.2. Deletion of crc Reduces the Expression of T3SS-Related Genes

**Figure 2:**
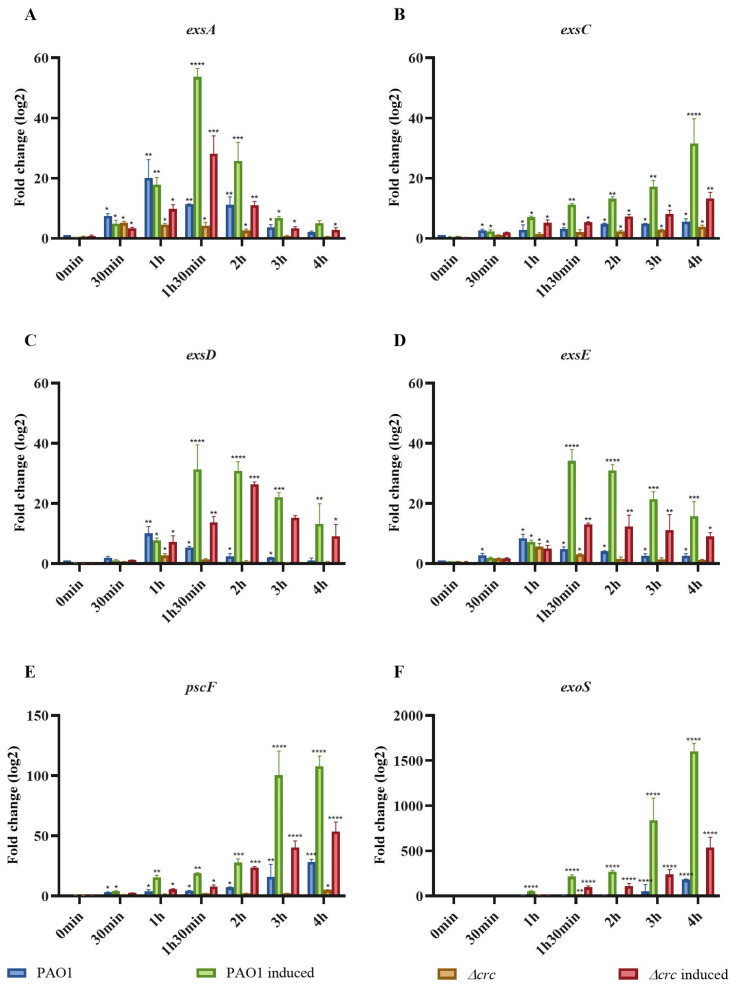
(**A**–**F**) The expression of T3SS associated genes is reduced in the *crc* mutant but their inducibility is preserved. The expression of genes involved in the regulation of T3SS expression (*exsA*, *exsC*, *exsD*, *exsE*), that codify proteins from the needle (*pscF*) and coding for the main secreted toxin (*exoS*) were analyzed by real time, quantitative PCR (RT-qPCR). Fold change values were calculated respect to levels of expression in PAO1 at time zero without induction. As shown, the expression of the studied genes was induced both in the wild-type strain and in the *Δcrc* mutant. However, the relative amount of all of them was lower, both under non-inducing and inducing conditions, in the mutant. Values that are significantly different by an unpaired two-tail *t*-test are indicated by asterisks as follows: * *p* < 0.1; ** *p* < 0.01; *** *p* < 0.001; **** *p* < 0.0001.

Time-courses of the expression levels of genes belonging to the T3SS regulon were measured in the PAO1 wild-type strain and the *crc* deficient mutant, under non-inducing and inducing conditions. In all cases and at all measured times, although the expression of T3SS-related genes was induced in both strains under inducing conditions, their level of expression was much lower in the *Δcrc* mutant than in the PAO1 wild-type strain, both under inducing and non-inducing conditions (Figure 2), being the differences between both strains statistically significant. As could be expected, *exsA*, encoding the master transcriptional regulator of the expression of the system, was the first to be induced. This reduced expression of T3SS-related genes may be causing a defective production of the T3S apparatus in the *crc* defective mutant.

### 2.3. T3S Is Impaired in the crc-Deficient Mutant, While the Intracellular Amount of T3S-Associated Proteins Remains Unchanged

**Figure 3:**
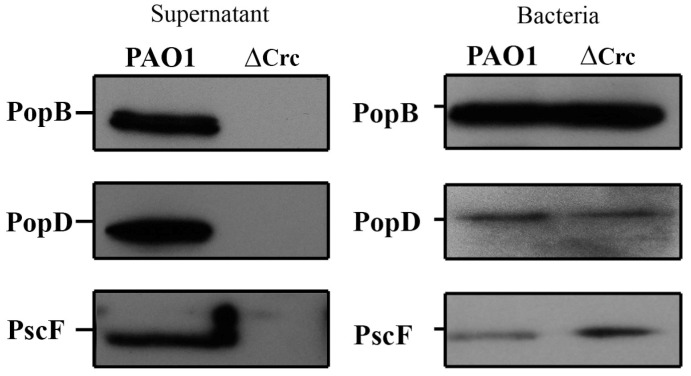
*crc* deletion impairs the extracellular accumulation of the proteins of the injectisome under T3SS-inducing conditions. Bacterial extracts and supernatants from PAO1 and *Δcrc* strains obtained on T3SS-inducing conditions were analyzed by Western blot using anti-PopB, anti-PopD and anti-PscF antibodies. Silver stained SDS-PAGE of these samples is shown in Supplemental Material Figure S1.

To compare the secretion profiles of the parental strain PAO1 and the *crc* deletion mutant, cells and supernatants from bacterial cultures grown on T3SS-inducing conditions were analyzed. In agreement with previous findings [10], the analysis of extracellular extracts showed that the overall secretion was similar in both strains, although the abundance of some secreted proteins was lower in the *crc* mutant that in the wild-type strain (Supplemental online Material Figure S1). Western blotting assays with polyclonal antibodies against the T3SS needle proteins (anti-PopB, anti-PopD, and anti-PscF) were performed to analyze the translocation of the proteins constituting the exportation apparatus. In agreement with previous findings [10], the secretion of the needle components under T3SS-inducing condition was completely abolished in the *Δcrc* mutant (Figure 3). However, the intracellular amount of PopB, PopD and PscF were close to that observed in the PAO1 wild type strain (Figure 3), a feature not analyzed before. These results suggest that an impaired secretion of the T3SS needle proteins might be involved in the defective phenotype of the *crc* deficient mutant.

### 2.4. ExsE Exportation Is Impaired in the crc-Deficient Mutant

**Figure 4:**
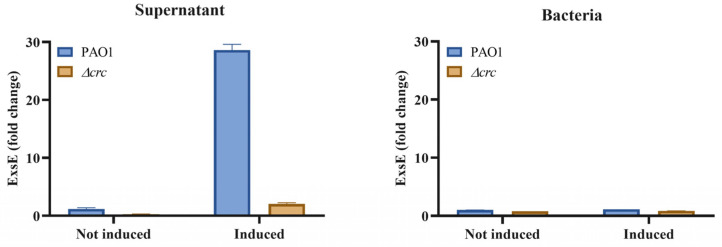
The secretion of ExsE is impaired in the *crc* deficient mutant. The amount of ExsE in bacterial extracts and supernatants of *P. aeruginosa* was measured by targeted quantitative proteomics. The Figure shows the fold change respect to the values of PAO1 under non-inducing conditions. As shown, the amount of secreted ExsE was lower, both under non-inducing and inducing conditions, in the *crc* mutant. Errors bars are given as standard deviation of the means of three biological replicates. The difference of secreted ExsE between the wild-type and the *crc* mutant was statistically significant under inducing and not inducing conditions (*p* < 0.05 calculated by unpaired two tail *t*-test).

As previously mentioned, the induction of the expression of the genes encoding the *P. aeruginosa* T3SS requires a complex regulatory cascade in which ExsE exportation through the T3SS needle is the first step in its activation [33,42]. To address if ExsE exportation is deficient in the *crc* mutant, and this deficiency could be the cause of the observed phenotype, its presence in the bacterial supernatants was determined. As shown, the amount of ExsE secreted protein was much lower in the *crc* mutant than in the wild-type strain (Figure 4). Together with the results shown in Figure 3, this result further supports that a sub-optimal activity of the T3SS in the *crc* mutant might be in the basis of its deficient T3S.

### 2.5. The Lack of Crc Impairs P. aeruginosa Proton Motive Force

**Figure 5:**
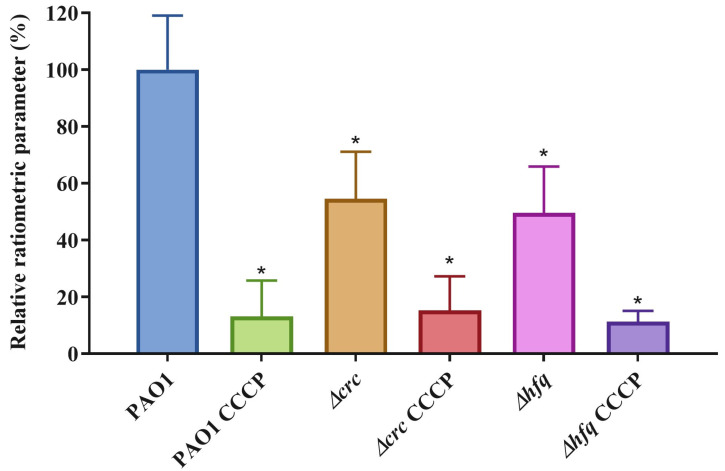
Deletion of *crc* disturbs *P. aeruginosa* proton motive force. Determination of the PMF in PAO1 and its *crc* and *hfq* deletion derived mutants. As shown, PMF of the *Δcrc* mutant was impaired in a similar degree observed for *hfq,* which was used as a control of deficient PMF, since it has been previously described to have a reduced membrane potential [44]. As a second control of impaired PMF, membrane depolarization was elicited with the addition of CCCP in the three strains. The experiments were performed in triplicates. Errors bars are given as standard deviation of the means of the biological replicates. * Indicates *p* < 0.05 calculated by unpaired two-tail *t*-test.

It has been previously proposed that PMF is required for the activity of the T3SS and hence, when PMF is impaired the translocon cannot secrete its substrates [40,41]. It has also been established that Crc is a key element in keeping *P. aeruginosa* metabolic robustness [8]. In the absence of this metabolic regulator, *P. aeruginosa* presents an increased respiration rate, linked to a metabolic reorganization that leads to impaired redox homeostasis, and probably, some degree of uncoupling in the oxidative phosphorylation [8]. All these changes in energy metabolism might be causing an impaired PMF in the *Δcrc* mutant. In favor of this possibility is the finding that an *hfq* deletion mutant, lacking the chaperone that regulates *P. aeruginosa* catabolic repression together with Crc [43], presents an impaired PMF [44]. It might be then possible that the impaired T3SS of the *crc* defective mutant might be due to a defect in PMF. To address this possibility, the PMF was measured in the *crc* defective mutant and its parental wild-type strain PAO1. In addition, an *hfq* deletion mutant was constructed, as described in Materials and Methods, to be used as a control, and its PMF was also determined. The PMF of the *Δcrc* mutant presented a 54.5% reduction compared with the *P. aeruginosa* wild type strain and the *Δhfq* –control- mutant presented a 49.6% reduction (Figure 5). These results support that the impaired PMF associated with Crc inactivation might have a relevant role in the deficient T3SS of the *crc* defective mutant. To further confirm the effect of Crc in PMF, and although the *Δcrc* mutant does not present any further mutation besides the *crc* deletion [11], the mutant was complemented by introducing an expression plasmid carrying the *crc* gene. As shown (Supplemental Material, Figure S2), the expression in trans of *crc* restored the PMF of the *Δcrc* mutant to the levels of the wild-type strain.

These results support that the impaired PMF associated with Crc inactivation might be on the basis of the observed impaired T3SS of the *crc* defective mutant.

## 3. Discussion

It has been previously discussed that bacterial virulence may be interlinked with common elements of microbial physiology and metabolism [45,46]. In that way, metabolic robustness has been considered a key property that promotes pathogenic fitness upon infection by allowing bacterial pathogens to address the nutritional changes and the stressing conditions that they can encounter in the infected host [47,48]. In the case of *P. aeruginosa*, previous work has shown that Crc may be a key element in mediating this crosstalk [10] at different levels, including the regulation of the production of virulence determinants [49], such as the T3SS [10], the modulation of secretion and production of extracellular vesicles [11], and the response to oxidative stress, which is linked to metabolic robustness [8]. The fact that the in vitro expression of the T3SS is triggered just under some specific growing conditions, as calcium deprivation, which strongly modifies bacterial metabolism, supports that the activity of this virulence determinant is interlinked with bacterial metabolism. Further, mutants in genes encoding metabolic enzymes such as the pyruvate dehydrogenases genes, *aceB* and *aceA*, secrete lower amounts of T3SS-related proteins (ExoS, PopB, and PopD) and are unable to activate transcription of *exsA* in response to calcium depletion, a condition used for triggering T3S in vitro [36]. Furthermore, it has been also described that mutations in the genes coding for CbrAB, a two-component system implicated in the sensing and response of carbon/nitrogen imbalance, restore the defect in T3S caused by the overexpression of histidine utilization genes [37]. It has been as well described that metabolic changes associated with the overexpression MDR efflux pumps may be responsible for the T3S impairment observed in mutants over-producing the efflux pumps MexCD-OprJ and MexEF-OprN [50].

The T3SS regulon is composed by ≈40 genes organized within 10 transcriptional units, each one controlled by an ExsA-dependent promoter. These genes encode the secretion and translocation machinery, regulators of T3SS genes’ expression (ExsA, ExsD, ExsC, and ExsE), chaperons and effectors. In the current work, we found that Crc does not impair the inducibility of the T3SS at the transcriptional level. However, the level of expression of T3SS encoding genes was lower in the *crc*-defective mutant, both under non-inducing and inducing conditions. Notably, and while we did not observe huge changes in the intracellular amount of the studied T3SS proteins in the *crc* defective mutant, those proteins were not found in the extracellular fraction of the *crc* mutant. Among them, the ExsE low-level secretion of the *crc* defective mutant seems particularly critical, since secretion of this regulator is a primary event in the regulatory cascade that triggers the expression of the T3SS genes [33,42]. This result suggests that a defect in the activity of the injectosome, not just a reduced transcription of the T3SS genes, as previously suggested [38,39], might be in the basis of the impaired T3S of this mutant. When looking at the causes of this impairment, metabolic robustness, which is controlled by Crc [8], emerges as a potential need for preserving the activity of the T3SS, which is energetically costly. In favor of this possibility is the finding that the activity of the T3SS requires a proficient PMF [40,41]. Moreover, it has been shown that a mutant lacking Hfq, the partner of Crc in regulating *P. aeruginosa* catabolic repression [43,51,52], presents a reduced PMF [44]. Our results support that PMF is impaired in the *crc* defective mutant and, most likely, this impairment might be the cause of the reduced activity of the T3SS system in this mutant. This reduced activity may produce, in turn, a feed-back effect in the regulation of T3SS expression. Under non-inducing conditions T3SS is expressed at lower levels in the *crc* mutant than in the wild-type strains. When expression is triggered, it remains lower in the *crc* mutant, likely because this mutant presents a defective ExsE secretion. As a consequence, and although the T3SS is slightly induced in the *crc* mutant, it presents an overall impaired T3S.

It is worth mentioning that the *crc* defective mutant presents also an increased susceptibility to a wide range of antibiotics. Interestingly, while the genes that encode some antibiotic transporters, such as OprD or GlpT, present a higher expression in the *crc* mutant, which may justify the increased susceptibility to the antibiotics they transport (imipenem and fosfomycin, respectively), the reason for the increased susceptibility to other antibiotics in the *crc* deficient mutant remains obscure. Although this increased susceptibility could be due to a reduced production of multidrug efflux pumps, it has been shown that the expression of these intrinsic resistance determinants does not change in the *crc* defective mutant [10], a feature also found in the case of the *hfq* mutant [44]. Since the activity of the Resistance–Nodulation–Division efflux pumps [44,53,54], major contributors to *P. aeruginosa* intrinsic resistance to antibiotics, depends on the PMF, it might be possible that the observed increased susceptibility to several antibiotics of this mutant is due to an impaired PMF-dependent activity of the mentioned MDR efflux pumps, an hypothesis which study goes beyond the purposes of the current article.

Altogether, our results indicate that the correct activity of the T3SS in *P. aeruginosa* requires a robust metabolism and presents different layers of integration; from the regulation of the expression of the components of the system to their export, assembly, and right functioning of the translocation apparatus. The results here presented, together with previously published articles, indicate that bacterial virulence, as well as intrinsic resistance, is fully integrated in the common regulatory circuits of bacterial physiology, in general, and microbial metabolism, in particular. As suggested in the case of antibiotic resistance [44,55], this opens the possibility of developing metabolic interventions that will simultaneously reduce the resistance to antibiotics and the virulence of bacterial pathogens.

## 4. Materials and Methods

### 4.1. Bacterial Strains and Growth Conditions

The bacterial strains used in this study were the wild-type *P. aeruginosa* strain PAO1 (PAO001, Holloway strain) and its isogenic deletion mutants *Δhfq* (see below) and *Δcrc*. Whole-genome sequence of the later had previously shown that its genome does not contain any secondary mutation besides the deletion of *crc* [11]. The analysis of the transcriptome of this mutant further confirmed that the *crc* deletion was the only genomic change that it presents [49] (NCBI, access code PRJNA934266). Bacteria were grown at 250 rpm and 37 °C in LB (Lysogeny Broth) Lennox medium (Pronadisa, Madrid, Spain) unless otherwise indicated. 1 mM IPTG was added in the complementation experiments.

### 4.2. Δhfq Mutant Construction

A deletion mutant of *hfq* was constructed by homologous recombination into the wild-type PAO1 strain. Two 500 bp DNA regions upstream and downstream of *hfq* were amplified by PCR from PAO1, using the oligonucleotides described in Table 1. PCR products containing HindIII restriction sites were cloned into HindIII-digested and dephosphorylated pEX18Ap vector [56], and the resultant plasmids introduced by transformation into *E. coli* S17-1 [57]. Then, conjugation and mutant selection were performed as described elsewhere [56] using 350 µg/mL carbenicillin and 10% sucrose. The presence of the deletion was confirmed by PCR amplification of the corresponding genomic region and Sanger sequencing.

### 4.3. Complementation of the Δcrc Mutant

To complement the *crc* mutant, the plasmids pVLT35 (control, cloning vector) and pVLT35*crc* (containing the complete *crc* gene) previously described [10] were transferred to *P. aeruginosa* PAO1 and *Δcrc* by tripartite mating, as previously described [58]. The presence of the plasmids in isolated exconjugant colonies was confirmed by PCR with primers shown in Table 1.

### 4.4. Detection of ExoS Injection in HeLa Cells by Immunofluorescence

The human cervix carcinoma cell line HeLa was cultured in DMEM medium supplemented with antibiotics (penicillin 100 U/mL-streptomycin 100 μg/mL), L-Glutamine (2 mM), and 10% heat-inactivated fetal bovine serum (FBS) at 37 °C in a humidified atmosphere containing 5% CO_2_.

Cells were grown on glass coverslips onto a p24 plate in complete medium without antibiotics and incubated 24 h at 37 °C in a humidified atmosphere containing 5% CO_2_. Then, HeLa cells were incubated with *P. aeruginosa* PAO1 and *Δcrc* at a MOI (Multiplicity Of Infection; HeLa cell/bacteria ratio) of 50 during 3 h.

The coverslips were washed two times with PBS and fixed with 4% formaldehyde in PBS for 20 min at RT. After fixation coverslips were washed twice with PBS and cell membranes were permeabilized for 15 min with PBS containing 0.2% Tween-20 at room temperature. After two washes with PBS, coverslips were incubated with Binding Buffer (0.1% Saponin, 0.2% BSA in PBS) for 30 min and overlaid with 1/500 Anti-ExoS antibody for 1 h. Then, they were washed with PBS three times and incubated for 1 h with 1/1000 Alexa Fluor 488 anti-rabbit, 1/2000 Phalloidin-TRITC (Sigma-Aldrich, Burlington, MA, USA) and DAPI. Coverslips were washed three times with PBS and prepared for microscopy by mounting them on slides with anti-fading solution.

Digital images were captured using a fluorescence microscopy (excitation/emission BP 480/30 and BP 535/40, respectively) and UV filters (excitation/ emission BP 365/12 and long pass 397, respectively).

### 4.5. T3SS Induction, RNA Preparation and Real Time RT-qPCR

In vitro T3SS induction in *P. aeruginosa* strains was performed as described [50]. Overnight cultures were diluted to an OD600 of 0.1 in calcium-depleted medium containing 5 mM EGTA and 20 mM MgCl_2_ (T3SS induction conditions). This was considered as time 0 (t0) and samples were taken at different times after induction and processed as described next. Ten milliliters of culture were collected by spin down at 4 °C (7000 rpm) and frozen in dry ice. Total RNA was extracted with the RNeasy Kit (Qiagen, Venlo, The Netherlands). DNA was removed by treatment with the DNA-Free Kit (Applied Biosystems, Waltham, MA, USA). To check that no residual DNA was present in the RNA samples, PCRs were performed with primers rplU-fw and rplU-rv (Table 1). The cDNA was obtained by a High Capacity cDNA Reverse Transcription Kit (Applied Biosystems, Waltham, MA, USA), as indicated by the manufacturer. The expression level of T3SS-related genes was measured using the primers described in Table 1. The relative amount of mRNA was calculated using the 2^−ΔΔCt^ method [59]. Gene expression data were normalized using the housekeeping gene *rpoN*, using oligonucleotides described in Table 1. RT-qPCR was performed using a Power SYBR Green Kit (Applied Biosystems). In all cases, the mean values of relative mRNA expression obtained in three independent triplicate experiments were considered.

### 4.6. Electrophoresis and Western Blotting

T3SS-induced cultures of *P. aeruginosa* (10 mL) were centrifuged at 7000 rpm during 20 min to separate cells and supernatants. Cellular extracts were obtained after sonication in 1 mL of lysis buffer (10 mM Tris, 1 mM EDTA and 1 mM PMSF, pH8). Supernatants were filtered (pore size 0.2 μm) and concentrated to 1 mL (Amicon Ultra 3K, Millipore, Burlington, MA, USA). The concentration of secreted proteins can vary depending on the conditions and the strain studied. Indeed, the *crc* mutant presents a statistically significant reduction in extracellular proteins of 58 ± 1.7%, as compared with the wild-type strain. Consequently, the protein extracts analyzed in each case were adjusted to the number of cells as described by others studying *P. aeruginosa* T3S [17,60,61], separated by SDS-PAGE, 10% acrylamide gel, and transferred onto nitrocellulose membranes. The membranes were blocked with 5% nonfat milk and incubated with primary antibodies anti-PopB and anti-PopD kindly provided by Dr. I. Attree, anti-PscF (Protein A purified antibody, GenScript, Piscataway, NJ, USA) and anti-ExoS (Agrisera, Vännäs, Sweden). Secondary peroxidase conjugated antibody (Bio-Rad, Hercules, CA, USA) was added following chemiluminescence detection (Inmobilon Western, Millipore, Burlington, MA, USA). Chemiluminescence images were saved as TIFF files.

### 4.7. Parallel Reaction Monitoring Analysis for ExsE Quantification

Samples were digested with trypsin on STRAP columns (PROTIFI, Farmingdale, NY, USA), according to the protocol described by the manufacturer. Tryptic peptides were dried in a speed-vacuum system and resuspended at 100 ng/µL, according to QUBIT quantification (Thermofisher Scientific, Waltham, MA, USA). An amount of 500 ng of each sample was loaded online on a C18 PepMap 300 µm I.D. 0.3 mm × 5 mm trapping column (5 µm, 100 Å, Thermo Scientific) and analyzed by LC-ESI MSMS using a Thermo Ultimate 3000 RSLC nanoUPLC coupled to a Thermo Orbitrap Exploris OE240 mass spectrometer. Peptides were separated on a 15 cm × 75 µm RP C18 column in a 60 min long gradient at a 300 nL/min flow rate. The liquid chromatographic system was coupled via a nanospray source to the mass spectrometer. Targeted proteomics experiments were performed in PRM mode (parallel reaction monitoring) monitoring two ExsE specific peptides, IESISPVQPSQDAGAEAVGHFEGR (827.738 *m*/*z*, charge 3+) and LADGDGTPLEAR (607.804, charge 2+). Selection and extraction of each of the transition areas for quantitation, was carried out with the Skyline v21.2 software [62] following the usual criteria in the design of targeted proteomics experiments and using previous information obtained through shotgun proteomics assays.

### 4.8. Determination of the Proton Motive Force

Membrane potential was assayed following the protocol described in [44] with some modifications. PAO1, and its corresponding *crc* and *hfq* deletion mutants were diluted to an OD600 of 0.1 and grown in 20 mL LB at 37 °C for 4 h. The membrane potential was measured using the dye 3,3′-diethyloxacarbocyanine iodide [DiOC_2_(3)]. The cultures were diluted to an OD_600_ of 0.12 in buffer containing 100 mM Tris, 1 mM EDTA, and 80 mM NaCl (pH adjusted to 7.4 using HCl). DiOC_2_(3) was added to a final concentration of 1.5 μM. For depolarized controls, carbonyl cyanide 3-chlorophenylhydrazone (CCCP) was added to a final concentration of 25 μM. The samples were incubated for 30 min at room temperature in darkness and then analyzed on a Gallios Flow Cytometer (Beckman Coulter, Brea, CA, USA). DiOC_2_(3) fluorescence was measured using excitation at 488 nm and emission at 530 ± 30 nm (GC) and 650 nm (RC). The analysis of the data was performed with Kaluza. Membrane potential was distinguished by an increased red to green fluorescence ratio. The radiometric parameter (red-to-green ratio) was calculated according to the manufacturer’s instructions [RC − GC + 1.5 × (#channels per decade)] and normalized to the value of the wild type strain.

## Figures and Tables

**Table 1:** Oligonucleotides used in this work.

Oligonucleotide	Sequence	Utilization
***rplU*** **-fw**	CGCAGTGATTGTTACCGGTG	Check DNA contamination in RNA samples
***rplU*** **-rv**	AGGCCTGAATGCCGGTGATC	
**RT-*rpoN*-fw**	GCAGAAATACATGCATACG	Internal control gene for RT-PCR
**RT-*rpoN*-rv**	TGTGCCTCCAGTAAACCAG	
**RT-*exsA*-fw**	TCAAGGGGTTGAAGGAATTG	RT-PCR for *exsA*
**RT-*exsA*-rv**	TCCATGAATAGCTGCAGACG	
**RT-*exsC*-fw**	GTCACCCTGTTGCTGCTC	RT-PCR for *exsC*
**RT-*exsC*-rv**	ATCTGCGCATACAACTGGAC	
**RT-*exsD*-fw**	AACTGTTCCGCTGCGAGT	RT-PCR for *exsD*
**RT-*exsD*-rv**	TTTCCCACCAGCCATAGAC	
**RT-*exsE*-fw**	AATCGATTTCGCCGGTGC	RT-PCR for *exsE*
**RT-*exsE*-rv**	GATCGCCAGCCACGTT	
**RT-*pscF*-fw**	CGCAGATATTCAACCCCAAC	RT-PCR for *pscF*
**RT-*pscF*-rv**	ATCTTCTGCAGGATGCCTTG	
**RT-*exoS*-fw**	AGAGAGCGAGGTCAGCAGAG	RT-PCR for *exoS*
**RT-*exoS*-rv**	ATGCCGGTGTAGAGACCAAG	
**HindIII-*hfq*-ups-fw**	AAGCTTCGATGCGCTGCCCTGCGAGC	Amplification of the DNA flanking region upstream of *hfq*
***hfq*** **-ups-rv**	GCGGACTCCCGTCAAGCGTTGCTTTTGACATGTGCCGCACT	
***hfq*** **-down-fw**	AGTGCGGCACATGTCAAAAGCAACGCTTGACGGGAGTCCGC	Amplification of the DNA flanking region downstream of *hfq*
***HindIII-hfq-down-rv***	AAGCTTTGTGCGGCTCGACCGAGGGT	
pVLT35-fw	GCGGATAACAATTTCACACAGGA	Check complementation of *Δcrc*
pVLT35-rv	CTCATCCGCCAAAACAGCC	

## Data Availability

All data needed to evaluate the conclusions of this work are present in the main document and/or the Supplementary Materials. Transcriptome of the *crc* deletion mutant can be found at NCBI (access code PRJNA934266).

## Supplementary Materials

The following supporting information can be downloaded at: https://www.mdpi.com/article/10.3390/ijms241512304/s1.
