# Supplementary material for: Virulence and Metabolism Crosstalk: Impaired Activity of the Type Three Secretion System (T3SS) in a Pseudomonas aeruginosa Crc-Defective Mutant
Source: Int J Mol Sci. 2023 Aug 1;24(15):12304. doi: 10.3390/ijms241512304 (PMC10419072; doi:10.3390/ijms241512304)
Supplement: Supplementary file 1 [file ijms-24-12304-s001.zip › ijms-2541155-supplementary.pdf]

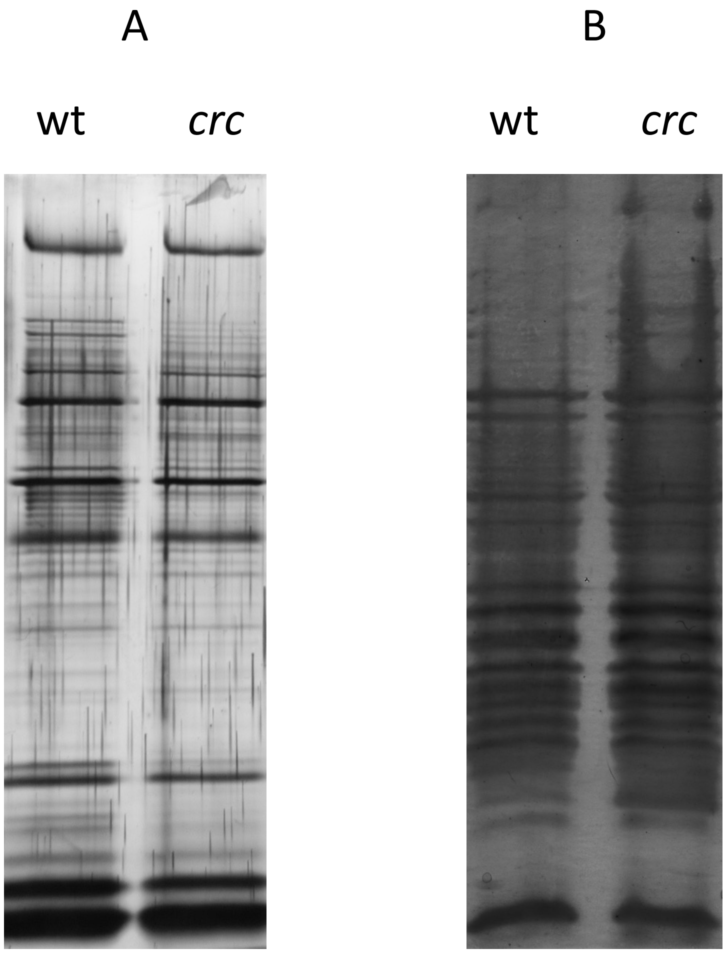

Figure S1. SDS-PAGE of samples used for Western blot. The gels were stained with silver. A: secreted proteins. B: intracellular proteins.

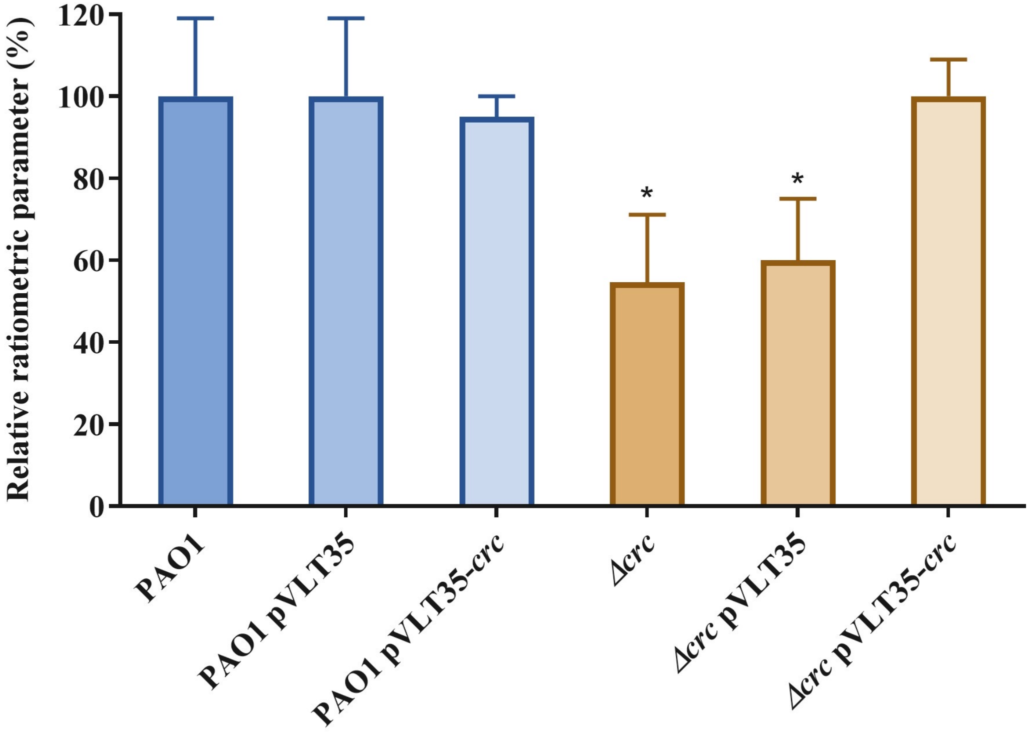

Figure S2. Complementatiom with *crc* restores the membrane potential of the Δ*crc* mutant. As shown, the trans-complementation of the Δ*crc* mutant with a plasmid-encoded *crc* restores the membrane potential to the levels of the wild-type strain. The experiments were performed in triplicates. Errors bars are given as standard deviation of the means of the biological replicates. * Indicates P < 0.05 calculated by unpaired two-tail t-test.
